# Miscellany

**Published:** 1904-10

**Authors:** 


### Miscellany.

REPAIRING FRACTURED CASTS.—A valuable method of repairing fractured plaster casts may be found in the use of celluloid dissolved in camphor and ether to a creamy consistence. A good quality of celuloid should be selected, and to it should be added a mixture of equal parts of ether and spirits of camphor. This combination dissolves celluloid rapidly, and should be added to the material until a solution of a cream consistence is obtained. The preparation should be kept tightly corked to avoid its evaporation.

When it becomes desirable to repair broken casts, the fragments to be attached should be well dried and both surfaces should be freed from broken particles. The surfaces should be coated with the celluloid solution, and after being pressed firmly, should be allowed to dry.—S. M. WEEKS.

---

TO PREVENT SORENESS OF THE LIPS AFTER OPERATING.—White perfumed vaseline will be found a most excellent preventative of sore lips from long and repeated applications of the rubber dam. Although many ladies object to its use on account of the general impression that it stimulates the growth of hair, I find upon explaining the good results from its use, the avoidance of the so-called cold sore, they will gladly submit to its application. Two covered porcelain jars with the pure white vaseline are kept in the operating-case, the contents of one for use as an application around the mouth, the contents of the other for strips and disks. By cleaning the porcelain jars every day, and placing in them a small quantity of the pure clean vaseline, they are ready for use, and if need be, the inspection of the most particular patient. It is the exception that the most fastidious lady, when handed the porcelain jar and the explanation is made for its use, will not apply it immediately, and later bless you for having suggested it. Undoubtedly the saliva saturating the skin and held there by the rubber plays an important part in addition to our pulling and stretching the mouth, in producing these disagreeable sores. In long operations, especially in molars and bicuspids, a second and third application sometimes is indicated, which can be made by loosening the dam from the holder, drying the corner of the mouth thoroughly with a napkin, then reapplying the vaseline. If applied to the lips when they are dry or cracked, it softens them to such an extent that much of the discomfort of an operation is avoided.—W. T. Chambers, Denver, Col.

---

Keep a Record of Your Treatments.—The various dental ledgers will probably meet the requirements necessary to record all permanent work, but a small book kept in a convenient place near the operator's chair will be found useful for recording the daily treatments. The busy dentist can not retain in his mind the condition of each tooth he treats temporarily, and the result is that he must practically diagnose every case when it is presented, no matter how often he has treated the tooth before. The examination form will at once show him which teeth require his attention, but it will not give him any aid in determining the condition of the teeth at the last sitting. Very often a dentist will remember the condition of an individual case, and it is right that he should. It will not always be necessary for the careful practitioner to rely solely upon records, but a record will show him the condition of the tooth at the last sitting, and he can instantly determine what course to pursue. It will take less time to record the treatment and prognosis than to make even a brief diagnosis when the patient calls for treatment. This method will also serve as a reference for the operator to determine the correct fees he should receive for each tooth. After the work is completed he can refer the patient to his record and charge a reasonable fee, which might otherwise seem exorbitant.—*Review.*

---

Method of tipping Bridge Teeth.—Prepare the tooth by grinding as is ordinarily done and burnish pure gold backing to position on the tooth, allowing the backing to project beyond the labial or buccal surface of the facing at the occlusal end. Hold the occlusal end of the tooth, with backing in position, against a flat surface having a right angle corner, such as a drawer in the bracket table, the labial surface of the facing being held even with the upper surface of the drawer. The projecting gold can now be turned down till it rests on the upper flat surface. Burnish the gold well against the facing and contour the tip if necessary. The case is now invested and 22-carat plate is flowed over the occlusal surface and tip as heavy as desired. After the crown is soldered grind away the gold that overlaps the edge in front, and this will produce a solid 22-carat tip. The same method can be adopted for bicuspids and molars in bridge-work. Flow 22-carat plate over the whole surface of the backing, making it heavy enough to protect the facing. Make lingual cusps, articulate, and attach with solder. —F. S. Miller, Warsaw, Wis.

---

Treatment of Contracture of Joints with Rontgen Rays. —Moser reports the excellent results obtained in two cases by radiation. Almost all the joints of the first patient were ankylosed, and there was palpable friction during movement,—probably the results of gout. A skiagram was taken of one knee and the patient complained that she had had pains in all her joints thereafter. Moser noticed that the knee that had been exposed seemed less swollen than before, and he applied the Röntgen rays as a therapeutic measure for a minute to each knee. The patient reported four days later that there had been marked improvement since, not only of the exposed joints, but of all the others. The improvement progressively continued until the patient is now able to dress herself and do up her hair, previously impossible, and take a half-hour walk.—*Centralblatt Chirurgie*, Leipsic.

